# A national study on nurses’ retention in healthcare facilities in underserved areas in Lebanon

**DOI:** 10.1186/1478-4491-11-49

**Published:** 2013-09-30

**Authors:** Fadi El-Jardali, Mohamad Alameddine, Diana Jamal, Hani Dimassi, Nuhad Y Dumit, Mary K McEwen, Maha Jaafar, Susan F Murray

**Affiliations:** 1 Department of Health Management and Policy, Faculty of Health Sciences, American University of Beirut, Riad El Solh, Beirut 1107 2020, Lebanon; 2 School of Pharmacy, Lebanese American University, Beirut, Lebanon; 3 Rafic Hariri School of Nursing, Faculty of Medicine, American University of Beirut, Beirut, Lebanon; 4 Alaska Department of Health and Social Services, Division of Public Health, Section of Health Planning and Systems Development, Juneau, Alaska; 5 Department of Health Policy & Management, Florence Nightingale School of Nursing & Midwifery, King’s College London, London, UK

**Keywords:** Nurses, Retention, Underserved areas, Hospital, Primary health care, Lebanon, Rural, Human resources for health

## Abstract

**Background:**

Nursing shortages and maldistribution are priority issues for healthcare systems around the globe. Such imbalances are often aggravated in underserved areas, especially in developing countries. Despite the centrality of this issue, there is a dearth of studies that examine the retention of nurses in underserved areas in the Middle East Region. This study investigates the characteristic and the factors associated with the retention of nurses working in rural areas in Lebanon.

**Methods:**

This study uses a non-experimental cross-sectional design to survey nurses working in underserved areas of Lebanon. Underserved areas in Lebanon were identified using WHO definition. A total of 103 health facilities (hospitals and primary healthcare centers) located in these areas were identified and all nurses working at these facilities received a copy of the survey questionnaire. The questionnaire included five sections: demographic, work-life, career plan, job satisfaction, and assessment of work environment. Analysis included univariate and bivariate (chi-square, Student’s *t*-test and ANOVA) tests to describe the respondents and examine the significance between nurses’ characteristics and their intent to stay. A logistic regression model was constructed to identify factors associated with nurses’ intent to stay in underserved areas.

**Results:**

A total of 857 nurses from 63 Primary Healthcare (PHC) centers and hospitals responded to the questionnaire (75.5% response rate). Only 35.1% of nurses indicated their intent to stay in their current job over the coming one to three years. Surveyed nurses were most satisfied with relationship with co-workers and least satisfied with extrinsic rewards. Rural nurses working in PHC centers were more satisfied than their hospital counterparts on all aspects of work and had significantly higher intention to stay (62.5% compared to 31.5% in hospitals, *P* < 0.001). Regression analysis revealed that nurses less likely to report intent to stay were younger, unmarried, with less years of work experience and were not working towards a higher degree. Analysis reveals a directly proportional relationship between nurses’ reported job satisfaction and their intent to stay.

**Conclusion:**

This study reveals poor retention of nurses in rural and underserved areas in Lebanon, especially in the hospital sector. The status quo is disquieting as it reflects an unstable and dissatisfied nursing workforce. Developing targeted retention strategies for younger nurses and those working in hospitals as well as the offering of professional development opportunities and devising an incentive scheme targeting rural nurses is pivotal to enhance nurses’ job satisfaction and retention in rural settings.

## Introduction

The maldistribution and shortages of human resources for health (HRH) in underserved areas is gaining the attention of both policy makers and researchers worldwide [1]. According to the World Health Organization (WHO), underserved areas include geographical areas where the population has limited access to quality health-care services and to qualified health-care providers [2]. WHO’s designated underserved areas may include: ‘remote and rural areas, small or remote islands, urban slums, conflict and post-conflict zones, refugee camps, minority and indigenous communities, and any place that has been severely affected by a major natural or man-made disaster’ [2] (page 9)*.* According to the WHO (2006), half of the world’s population resides in rural areas; however this population is served by only 38% of the global nursing workforce [3]. The inequitable distribution of HRH in rural areas is a major problem contributing directly to the global burden of disease and negatively influencing health outcomes in these areas [4]. This shortage is most pronounced for nurses who are the linchpin of a healthcare system and who are responsible for delivering the majority of healthcare to their communities [5].

A multitude of strategies, with varying degrees of success, have been implemented by governments to address this problem and attract nurses to underserved areas. These strategies include financial incentives, non-financial incentives, educational or recruitment interventions and regulatory interventions [2,6,7]. Within the context of the Eastern Mediterranean Region (EMR), there has been a dearth of studies that have systematically investigated the factors significantly associated with nurses’ retention in underserved areas.

### Retention of HRH in underserved/rural areas

Literature reports a global shortage of nurses compounded by geographic imbalances in the distribution of the nursing workforce [5,8,9]. Such shortage is particularly challenging in rural and remote areas since recruiting and retaining nurses in these areas has been shown to be more difficult [10,11]. Strategies such as increasing the number of graduates do not ensure that these graduates will choose to practice in underserved areas [8,12]. As such, there is a need to specifically explore the factors associated with the retention of nurses working in underserved areas to derive specific policy recommendations to meet their particular needs.

A model proposed by Henderson and Tulloch [12] and later adapted by WHO [2] detailed factors affecting nurses’ intent to stay in rural and remote areas. These factors are complex and multifaceted and can pertain to several dimensions such as personal aspects, family and community reasons, financial aspects, career issues, working conditions, living conditions, and mandatory service [2,12]. The interplay of these and other factors has also been cited in related literature, whereby individual factors including gender, age, marital status, rural or urban upbringing, area of education and original professional plan at the time of enrollment in educational programs have been documented as influencing career decisions. Economic considerations related to salary and benefits and total medical school debt are other determinants influencing intent to stay. Additional factors relate to organizational, institutional and socio-cultural environment such as personality and practice conflicts, workload, material availability and area lifestyle issues respectively [8,12-14].

### Local context

Lebanon is characterized by an oversupply of physicians and an undersupply of nurses. The country lacks clear and accurate audit of the actual stock of physicians, nurses and midwives, and annual supply of HRH from medical and nursing schools [15]. However, available data shows that approximately 6,000 nurses are working in Lebanon [15]. Nurses practicing in Lebanon have different types of degrees which can be technical (non-university) such as baccalaureate technique (BT), technique superieur (TS) or licence technique (LT) or a university degree, mainly a Bachelor of Science in Nursing (BSN) or Master of Nursing (MS). The Ministry of Public health updated the law governing the nursing profession (Decree 1655) to classify and define the role and scope of nursing professionals as ‘professional nurses’ holding TS, BSN or higher degrees; these nurses are equivalent to Registered Nurses. Nurses holding technical degrees such as BT and LT are termed ‘nurses’ and those who had one year’s worth of nursing training in a hospital or educational setting are labeled ‘assistant nurses’ [16]. Nurses with a BT or BSN are required to sit for national examinations governed by the Ministry of Education in order to qualify to practice in the nursing profession. TS nurses are not required to sit for this examination and this degree requires one additional year or study after LT [17].

Lebanon is a source country of HRH [18]. Many nurses choose to immigrate to countries of the Gulf, Europe and North America in search of better job opportunities. Nurse migration has reached alarming rates with recent estimates of one of every five nursing graduates migrating out of Lebanon within one to two years of graduation [18]. Furthermore, two thirds of currently employed Lebanese nurses reported intent to leave their jobs within one to three years; 36.7% of which disclosed plans to leave the country [19]. This high rate of nurse attrition is related to a number of challenges including professional and geographic misdistribution, out-migration, limited opportunities for continuing medical education programs and career development and limited financial and non-financial incentives [20].

Lebanon is also facing challenges pertaining to sectoral maldistribution whereby PHC centers have been facing difficulties recruiting and retaining health workers. A recent study focusing on retention of human resources in PHC centers found that two out of five respondents indicated intent to quit their job within a period of one to three years. Reported reasons for this intent to quit included poor salaries, availability of better employment opportunities abroad, lack of professional development, job instability and lack of managerial support. Respondents also exhibited signs of professional burnout, reporting high levels of emotional exhaustion, depersonalization, and low levels of personal accomplishment [21].

### Objectives

This study aims at describing the current status and characteristics of nurses working in underserved areas in Lebanon and identifying the factors associated with nurses’ retention in underserved areas with comparison across the sector of employment (hospital versus PHC).

## Methods

### Design

This non-experimental quantitative study utilizes a cross sectional research design to understand factors significantly associated with nurses’ retention in underserved areas in Lebanon. This study is part of a four-country study conducted in Yemen, Jordan, Lebanon and Qatar examining nurse staffing in underserved areas [22,23].

### Ethical approval

Ethical approval was obtained from the WHO Ethical Review Committee (ERC) (Protocol number RPC312) and Institutional Review Board (IRB) of the American University of Beirut (Protocol number FHS.FE.06).

### Identification of underserved facilities

Underserved areas in this study were identified based on the previously mentioned WHO definition. Based on this definition, indicators (educational, health, economic, infrastructure and so on) were extracted and compiled in data sheets and ranked to allow researchers to determine which areas could be classified as underserved compared to other regions. Some of the indicators used included distribution of health workers (specifically nurses and their educational qualifications by region), number of hospitals, health coverage, population distribution, educational levels, household expenditure, availability of drinking water and electricity. Since there are no international cut-off points for classification of underserved areas, the researchers identified these areas by comparing the aforementioned indicators across all Lebanese regions. The lowest ranking regions on these indicators were the selected ones (North, South and Bekaa). The researchers validated their choice with stakeholders from the Ministry of Health and the Order of Nurses. Based on these indicators, a total of 103 health facilities (hospitals and PHCs) in Lebanon were identified and all nurses working at these facilities were targeted.

### The data collection instrument

The data collection instrument was developed after a thorough review of the literature pertaining to the study objectives. It included several sections addressing different aspects of the nursing profession with a specific focus on those working in underserved areas. These sections were: demographic information (gender, age, marital status, nationality, having children, area of residence, living preferences (city or village), highest nursing credential and so on), work life (employment status, monthly income and receiving salary on time, additional benefits, standard of living, total working experience, working at previous facilities and so on), career plans (intent to stay, working towards higher nursing credentials, participating in continuing education, attitudes about nursing and so on), job satisfaction (using the McCloskey Mueller Satisfaction Scale), assessment of work environment (included 14 questions, some of which were selected from the Revised Nurse Working Index (NWI-R) developed by Aiken and Patrician (2000)), and hospital work (only used in the context of hospitals and included questions related to type of shift, clinical unit and nursing model). A combination of close-ended and open-ended questions were used to obtain the information needed. The questionnaire included the components of the aforementioned WHO adapted conceptual model developed by Henderson and Tulloch [12]. The model included five dimensions: (1) personal or general characteristics, (2) values and altruism (attitudes about nursing), (3) family and living conditions, (4) career and work related issues (measured using the McCloskey Mueller Satisfaction Scale - MMSS), and (5) financial incentives. The main outcome measure for the analysis was nurses’ decisions to stay in their post in the next one to three years.

It should be noted that the version of the MMSS included in this study reflects 25 of the 31 original items [24]. Six items were removed as they were not relevant to these nurses’ work context in rural areas in Lebanon. These items related to interaction with faculty members at schools of nursing, opportunities to participate in nursing research, opportunities to write and publish, control over work conditions, and control over what goes on in their work settings. Items were rated on a four-point Likert scale (ranging from ‘very dissatisfied - score of 1’ to ‘very satisfied - score of 4’). The revised version of the MMSS has been previously validated in a national study in Lebanon [19]. Nurses were asked to indicate their intent to stay (outcome variable) by specifying the likelihood of remaining in their current job for the foreseeable future. Answers were grouped into stay or leave depending on the reported likeliness to stay.

The survey instrument was originally developed in English and translated to Arabic by a professional translator. Back translation was conducted by two members of the research team to account for some context specific corrections to wording and phrasing of questions. Cognitive interviewing of the content of the questionnaire was conducted with a total of nine nurses to allow further corrections to the content, wording and flow of the questions in the questionnaire [25,26]. Nurses were asked to first review the English version of the questionnaire and comment on its content. A week later, they were asked to review the Arabic version of the questionnaire and also to report if any changes in content or wording were required. A period of two months was required for cognitive interviewing as the questionnaire was subject to several phases of modification before it was finalized. Three of the interviews were conducted first, then changes were made and a separate group of three nurses conducted the second phase of the interviews during which additional changes were recommended. The final stage was conducted with three other nurses and yielded minimal changes after which the surveys were finalized.

### Data collection

Facility directors at the 103 facilities were approached via a formal letter explaining the purpose of the study. Upon obtaining their approval for participation, they were sent the study questionnaire and were asked to hand them out to nurses working at their facility. Questionnaires were distributed in envelopes and directors were asked to give nurses time to respond and remind them to return them should they wish to participate. The survey questionnaire included a consent form on the first page which offered an overview of the research project and its objectives and requested participating nurses to consent to participation by ticking a box without specifying their name. Respondents were asked to complete the questionnaires at a place where they can have privacy and then put the questionnaire in the envelope and seal it prior to returning it to the facility director’s office so as to maintain the confidentiality of their responses. The research team promptly picked up all completed envelopes from all facilities.

### Data management and analysis

Information in the returned questionnaires was entered into an interface designed using the program CSPro 4.0. This program allows the development of algorithms to detect out-of-range values and alert the user to data discrepancies. The program also allows for auditing and correcting entered information.

Data was analyzed using the Statistical Package for Social Sciences Software (SPSS 19.0) for analysis (significance level 0.05). Univariate descriptive analysis was conducted to summarize demographic and work characteristics of responding nurses. Chi-square test was then used to compare nurses’ characteristics by their intent to stay and location of practice. Intent to stay was defined as desire to remain in their current job for the next three years. The literature defines intent to stay as ‘nurse’s plan in projected number of years to remain active in the nursing profession in practice, education, administration, or research’ [27].

A comparison of means using Students’ sample *t*-test was used to compare nurses’ characteristics with their intent to stay and location of practice. A logistic regression model was then constructed to better understand determinants of intent to stay for all respondents and across locations of practice. Questions included in the regression model were selected based on whether they were statistically significant at the bivariate level. Some questions (particularly age) were removed to eliminate collinearity and to obtain a better model that can explain intent to stay according to location of practice.

## Results

A total of 103 facilities were approached; 71 initially consented to participate but 8 did not return the surveys, yielding a 61% facility response rate (63 of 103 facilities). Out of a total of 1,135 nurses working in these 63 facilities a total of 857 nurses responded to the questionnaire (75.5% response rate). *P*-values in the bivariate analysis have been labeled ‘*p*’.

### Nurses’ characteristics and their intent to stay

Overall, only 35.1% of nurses indicated that they were likely or very likely to stay in their current job for the coming one to three years (See Table 1). Analysis showed that the majority of respondents were females (80%). Most responding nurses were below 30 years of age (58%) and this age group was least likely to report an intent to stay in current job (27.5%) (Table 1). A total of 33.3% of responding nurses were married with children and this group was more likely to report an intent to stay (45%). A total of 82% of responding nurses were raised in rural areas and around 80% reported holding non-university degrees. Almost half of the nurses (49%) had less than five years of overall working experience and this group was least likely to report an intent to stay (28.1%). Overall, 88.7% of the respondents worked in a hospital while only 11.3% worked at PHC centers. Nurses working at a PHC center were significantly more likely to report intent to stay (See Table 1).

**Table 1:** Nurses characteristics stratified by their intent to stay

				**Intent to stay**		
		**N**	**%**	**N**	**%**	***P*-value**
Intent to stay in job in coming 1 to 3 years	Stay	293	35.1	-	-	
	Leave	542	64.9	-	-	
Gender	Female	675	80.0	240	36.4	0.106
	Male	169	20.0	49	29.7	
Age	< 30 years	486	58	131	27.5	< 0.001
	30 to 45 years	311	37.1	128	42.2	
	≥ 46 years	41	4.9	27	69.2	
Family status	Married with children	273	33.3	117	44.8	< 0.001
	Married without children	117	14.3	47	40.5	
	Never married	429	52.4	110	26.1	
Rural upbringing	Yes	665	82.0	220	34.0	0.264
	No	146	18.0	60	41.7	
Highest degree institution	University	167	20.6	51	31.1	0.223
	Non-university	642	79.4	227	36.2	
Type of degree	Technical	778	92.5	270	35.5	0.491
	University	63	7.5	19	31.2	
Length of professional work as a nurse	< 5 years	420	49.0	116	28.1	< 0.001
	5 to 10 years	271	31.6	81	30.8	
	11 to 15 years	70	8.2	38	54.3	
	> 15 years	96	11.2	58	65.2	
Working towards higher degree?	Yes	282	33.6	73	26.5	< 0.001
	No	558	66.4	220	40.2	
Place of work	Hospital	760	88.7	233	31.5	< 0.001
	PHC	97	11.3	60	62.5	
Attitudes about nursing (Yes/No questions, responses to the option ‘Yes’ are reported)						
My family has a positive view about nursing		725	87.3	266	37.2	0.001
Other health professionals have a positive perception about nurses		589	73.0	221	38.2	0.003
My community has a positive image of the nursing profession		539	65.6	220	41.5	< 0.001
Most parents in this community would encourage their children to consider nursing as a career		342	41.9	143	42.6	< 0.001
I would encourage my nursing peers in other areas to move to my place of employment		348	40.6	161	47.2	< 0.001
Given the opportunity to choose my career all over again, I would choose nursing		264	30.8	129	50.4	< 0.001
I would encourage my daughters to consider nursing as a career		151	18.9	77	52.4	< 0.001
I would encourage my sons to consider nursing as a career		133	16.4	66	50.8	< 0.001

Survey respondents had mixed attitudes about their profession. While, the majority indicated that their families (87.3%) and other health professionals (73.0%) had positive attitudes about the nursing profession and 65.6% indicated that the community they were working in had a positive image about the nursing profession, only 30.8% reported that they would choose nursing as a profession if they could choose their career all over again. Moreover, very few indicated that they would encourage their daughters (18.9%) and sons (16.4%) to consider nursing as a career (See Table 1).

### Intent to stay and location of practice

To investigate whether nurses’ intent to stay and the factors associated with it differ by work setting, we compared the responses of nurses working in the hospital sector with those working in the PHC sector (Table 2). With respect to nurse demographics, analysis reveals that male nurses were significantly more likely to work in hospitals rather than PHC centers (22% compared to 4.2% respectively, *P* < 0.001). Younger nurses (< 30 years) tended to dominate the workforce in hospitals (61.2%) whereas nurses in the 30 to 45 years age group comprised the majority of those working in PHC centers (53.7%). ‘Never married’ nurses were more likely to work in hospitals (55%) whereas married nurses with children were more likely to work in PHC centers (58.7%, *P* < 0.001). Comparing nurses across professional characteristics reveals that nurses with technical degrees comprised the majority of the workforce in both hospitals and PHC centers, but nurses holding university degrees were significantly more likely to work in hospitals (8.3%, *P* = 0.01). While less experienced nurses (< 5 years) were more likely to report hospital employment (51.2%), more experienced nurses (> 15 years) were more likely to work in PHC centers (34%, *P* < 0.001) (Table 2).

**Table 2:** Comparing nurses’ characteristics by location of practice

		**Hospital**		**PHC**		***P*-value**
		**N**	**%**	**N**	**%**	
Gender						
	Female	584	78.0	91	95.8	< 0.001
	Male	165	22.0	4	4.2	
Age						
	< 30 years	455	61.2	31	32.6	< 0.001
	30 to 45 years	260	35.0	51	53.7	
	≥ 46 years	28	3.8	13	13.7	
Married with children						
	Married with children	219	30.1	54	58.7	< 0.001
	Married without children	108	14.9	9	9.8	
	Never married	400	55.0	29	31.5	
Rural upbringing?						
	Yes	598	83.2	67	72.8	0.015
	No	121	16.8	25	27.2	
Highest degree institution						
	University	147	20.5	20	22.0	0.738
	Technical	571	79.5	71	78.0	
Type of degree						
	Technical	686	91.7	92	98.9	0.013
	University	62	8.3	1	1.1	
How long have they worked professionally as a nurse						
	< 5 years	389	51.2	31	32.0	< 0.001
	5 to 10 years	247	32.5	24	24.7	
	11 to 15 years	61	8.0	9	9.3	
	> 15 years	63	8.3	33	34.0	
Intent to stay in your current job for the foreseeable future (the coming 1 to 3 years)?						
	Stay	233	31.5	60	62.5	< 0.001
	Leave	506	68.5	36	37.5	
Working towards a higher degree						
	Yes	261	34.9	21	22.6	0.017
	No	486	65.1	72	77.4	
Attitudes about nursing (these were a series of Yes/No questions, responses to the option ‘Yes’ are reported)						
	My community has a positive image of the nursing profession	463	63.6	76	80.9	0.004
	Other health professionals have a positive perception about nurses	508	71.1	81	87.1	0.004
	My family has a positive view about nursing	636	86.3	89	95.7	0.032
	Most parents in this community would encourage their children to consider nursing as a career	293	40.5	49	53.3	0.051
	I would encourage my sons to consider nursing as a career	110	15.2	23	25.6	0.042
	I would encourage my daughters to consider nursing as a career	117	16.5	34	37.8	< 0.001
	Given the opportunity to choose my career all over again, I would choose nursing	212	27.9	52	53.6	< 0.001
	I would encourage my nursing peers in other areas to move to my place of employment	286	37.6	62	63.9	< 0.001

Overall, nurses working in PHC centers were more likely to report intent to stay compared to nurses working in hospitals (62.5% versus 31.5% respectively, *P* < 0.001) (Table 2). In addition, nurses working in PHC centers generally had more positive attitudes towards nursing. The majority indicated that their community (80.9%, *P* = 0.004) and other health professionals (87.1%, *P* = 0.004) had a positive image of the profession. They also indicated that their families had a positive view of the profession (95.7%, *P* = 0.032) but few would encourage their sons (25.6%, *P* = 0.042) and daughters (37.8%, *P* < 0.001) to consider nursing as a career. Those nurses also indicated that they would encourage their nursing peers to move to their place of employment (63.9%, *P* < 0.001) but despite this a bit more than half reported that they would consider nursing as a career if they were given the choice to make all over again (53.6%, *P* < 0.001) (Table 2).

### Understanding the link between satisfaction, intent to stay and location of practice

Analysis of the MMSS revealed that the overall satisfaction score for survey respondents was 2.5 (±0.5) (reminder MMSS scores range from 1 to 4). Nurses were least satisfied with extrinsic rewards (2.1 ± 0.7) and most satisfied with co-workers (3.1 ± 0.5). Nurses who reported intent to stay had significantly higher satisfaction scores compared to nurses with intent to leave (Table 3). Specifically, nurses expressing intent to stay were most satisfied with access to professional development opportunities (3.2 ± 0.5), interaction opportunities (2.89 ± 0.55) and degree of control and responsibility (2.9 ± 0.5). On the other hand, nurses expressing intent to leave were least satisfied with extrinsic rewards (2.0 ± 0.6), work scheduling (2.3 ± 0.7) and balance of family and work (2.3 ± 0.6).

**Table 3:** Comparison of responses on MMSS with nurses’ intent to stay and location of practice

		**Intent to stay or leave**			**Location of practice**		
		**Stay**	**Leave**		**Hospital**	**PHC**	
**MMSS scale**	**Mean (SD)**	**Mean − SD − Median**	**Mean − SD − Median**	***P*-value**	**Mean − SD − Median**	**Mean − SD − Median**	***P*-value**
Overall satisfaction	2.5 (0.5)	2.7 − 0.4 − 2.8	2.4 − 0.4 − 2.5	< 0.001	2.5 − 0.4 − 2.5	2.8 − 0.5 − 2.9	< 0.001
Extrinsic rewards	2.1 (0.7)	2.4 − 0.7 − 2.3	2.0 − 0.6 − 2.0	< 0.001	2.0 − 0.6 − 2.0	2.5 − 0.7 − 2.7	< 0.001
Balance of family and work	2.2 (0.6)	2.7 − 0.5 − 2.8	2.3 − 0.6 − 2.4	< 0.001	2.3 − 0.7 − 2.5	2.7 − 0.7 − 3.0	< 0.001
Scheduling	2.4 (0.6)	2.5 − 0.7 − 2.6	2.3 − 0.7 − 2.3	< 0.001	2.4 − 0.6 − 2.3	2.8 − 0.5 − 3.0	< 0.001
Professional opportunities	2.5 (0.7)	3.2 − 0.5 − 3.0	3.0 − 0.5 − 3.0	< 0.001	3.1 − 0.5 − 3.0	3.3 − 0.5 − 3.0	< 0.001
Control and responsibility	2.6 (0.6)	2.9 − 0.5 − 3.0	2.7 − 0.6 − 2.8	< 0.001	2.7 − 0.5 − 2.8	3.0 − 0.3 − 3.0	< 0.001
Praise and recognition	2.7 (0.6)	2.7 − 0.6 − 3.0	2.4 − 0.7 − 2.0	< 0.001	2.4 − 0.7 − 2.0	2.8 − 0.6 − 3.0	< 0.001
Interaction opportunities	2.7 (0.5)	2.9 − 0.6 − 3.0	2.5 − 0.7 − 2.5	< 0.001	2.6 − 0.6 − 2.8	2.9 − 0.7 − 3.0	0.001
Co−workers	3.1 (0.5)	2.8 − 0.6 − 3.0	2.4 − 0.7 − 2.5	< 0.001	2.5 − 0.7 − 2.5	2.8 − 0.6 − 3.0	< 0.001

Satisfaction scores were also significantly different across locations of practice. Scores on all subscales were significantly higher for nurses working in PHC centers and were highest for interaction opportunities (2.9 ± 0.7), praise and recognition (2.8 ± 0.6), and scheduling (2.8 ± 0. 5). Similar to nurses expressing intent to leave, hospital nurses were least satisfied with extrinsic rewards (2.0 ± 0.6), balance of family and work (2.3 ± 0.7) and work scheduling (2.4 ± 0.6) (Table 3).

### Determinants of intent to stay

Results from the regression model revealed that married nurses had greater odds of expressing an intent to stay in their current job with higher odds reported to married nurses with children (odds ratio (OR) = 1.8 and 2.0 respectively). Marriage was found to be significantly associated with intent to stay for nurses working in hospitals but not for those working in PHC centers.

Compared to nurses with more than 15 years of experience, nurses with more than 5 years of experience had 0.3 (95% CI = 0.1 to 0.6; *P* = 0.001) the odds of expressing intention to stay and those with 5 to 10 years of experience had 0.3 (95% CI = 0.1 to 0.5; *P* < 0.001) the odds of expressing intention to stay. This observation was similar for nurses working in hospitals, yet with even smaller odds (Table 4).

**Table 4:** Revised Regression Model to understand determinants of intent to stay

		**Overall**			**Hospitals**			**PHC**		
		**OR**	**95% CI**	***P*-value**	**OR**	**95% CI**	***P*-value**	**OR**	**95% CI**	***P*-value**
Gender										
	Female	1			1			1		
	Male	1.0	0.7 to 1.6	0.971	1.0	0.6 to 1.6	0.93	2.6	0.1 to 55.2	0.540
Age										
	< 30 years	0.6	0.2 to 1.5	0.253	1.4	0.4 to 4.7	0.638			
	30 to 45 years	0.5	0.2 to 1.4	0.211	1.4	0.4 to 4.4	0.587			
	≥ 46 years	1			1					
Married with children										
	Married with children	1.8	1.1 to 2.7	0.010	2.0	1.2 to 3.2	0.005	0.4	0.1 to 1.9	0.255
	Married without children	2.0	1.2 to 3.3	0.011	2.3	1.3 to 4.0	0.003	0.8	0.1 to 7.6	0.837
	Never married	1			1			1		
Total working experience as a nurse										
	< 5 years	0.3	0.1 to 0.6	0.001	0.2	0.1 to 0.4	< 0.001	0.3	0.1 to 2.1	0.242
	5 to 10 years	0.3	0.1 to 0.5	< 0.001	0.2	0.1 to 0.4	< 0.001	0.2	0.03 to 1.2	0.074
	11 to 15 years	0.7	0.3 to 1.7	0.489	0.4	0.6 to 1.1	0.085	1.2	0.1 to 14.3	0.883
	> 15 years	1			1			1		
Are you currently working towards a higher degree										
	No	1.8	1.2 to 2.6	0.006	1.5	1.0 to 2.3	0.076	11.5	2.4 to 55.3	0.002
	Yes	1			1			1		
I would encourage my sons to choose nursing as a career										
	No	1			1			1		
	Yes	1.8	1.1 to 2.8	0.016	2.0	1.2 to 3.4	0.007	0.6	0.2 to 2.4	0.497
I would encourage my nursing peers in other areas to move to my place of employment										
	No	1			1			1		
	Yes	2.0	1.4 to 3. 0	0.001	1.6	1.1 to 2.5	0.024	21.8	3.5 to 136.8	0.001
Overall satisfaction		4.5	2.8 to 7.2	< 0.001	5.3	3.1 to 9.1	< 0.001	1.3	0.3 to 5.7	0.700

Respondents who indicated that they were not working towards a higher degree had 1.8 the odds of expressing intention to stay (95% CI = 1.2 to 2.6; *P* = 0.006). This observation was of particular significance for nurses working in PHC centers where the odds of intent to stay were 11.5 (95% CI = 2.4 to 55.3, *P* = 0.002).

Nurses who indicated that they would encourage their sons to consider nursing as a career had 1.8 times the odds to express intention to stay (95% CI = 1.1 to 2.6; *P* = 0.006). This observation also held true for nurses working in hospitals where the odds of intent to stay were 2.0 (95% CI = 1.2 to 3.4, *P* = 0.007).

Nurses who reported that they would encourage their nursing peers to move to their place of employment also had greater odds of expressing intention to stay (OR = 2.0, 95% CI = 1.1 to 3.0; *P* = 0.01) (Table 4). This observation was significant for both hospitals and PHC centers where the former had 1.6 higher odds (95% CI = 1.0 to 2.5, *P* = 0.02) of intent to stay and the latter had 21.8 higher odds (95% CI = 3.5 to 136.8, *P* = 0.001) of intent to stay (Table 4).

Higher scores on overall satisfaction were also associated with higher odds of staying (OR = 4.5, 95% CI = 2.8 to 7.2; *P* < 0.001). Overall satisfaction was significantly associated with intent to stay in hospitals but not with intent to stay in PHC centers (OR = 5.3, 95% CI = 3.1 to 9.1, *P* < 0.001) (Table 4).

## Discussion

This study was the first of its kind in both Lebanon and the EMR to investigate the retention of nurses working in underserved areas. Findings from this study revealed that several demographic and personal characteristics of nurses were associated with their intent to stay in their current job.

Study findings revealed that nurses less likely to report intent to stay had fewer years of experience and were thus younger. The fact that age was only significantly associated with intent to stay at the bivariate level and not significant at the multivariate level could be attributed to the fact that age and years of experience go in parallel; more experienced nurses are typically older. The direct association between years of experience/age and intent to stay is consistent with other studies in literature [28,29]. Our findings prompt nurse planners, policy and decision makers to design and implement targeted retention initiatives aimed at retaining new nurse graduates, less experienced and younger nurses in rural and remote communities [30]. Such initiatives must be designed in an engaging approach to involve younger and less experienced nurses in planning and implementation [7].

Study findings further indicated that married nurses and those married with children were twice as likely to indicate intention to stay in their current job as compared to their unmarried counterparts. Such findings are also in accordance with previously reported studies in the literature [31]. Sociocultural factors related to the perceived role of married women in society may also play a role in enhancing married nurses’ job retention. Most married females in Lebanon (and other Middles Eastern countries) are expected to take the lead in terms of caring for their families and children [32]. They do thus prefer job stability close to their homes and their children’s schools. They are also less mobile compared to their single counterparts since married nurses with children may find it difficult to relocate with the whole family and have a higher sense of financial responsibility [33]. They generally also display higher levels of job commitment [34].

Analysis of the MMSS reveals that surveyed rural nurses were most satisfied with the ‘relationship with co-workers’ aspect of work. Such a relationship appears to be an important motivator that is enhancing rural nurses’ retention and possibly mitigating the negative aspects of employment in rural and remote communities. Furthermore, encouraging peers to move to the nurse’s place of employment is also indicative of significantly higher odds of staying in current job. A strong relationship with co-workers has been found to be important in modern healthcare systems and has been linked to improved work environment which has a critical impact on patient safety. Managers of rural health facilities are encouraged to organize programs and activities that strengthen team work and enhance professional collaboration. Such programs are found to not only enhance the quality of patient care but also the retention of nurses [35,36].

Study findings revealed a clear issue with motivating and rewarding nurses with extrinsic rewards. There is overwhelming evidence in the literature that motivating nurses would increase their job satisfaction and positively influence their intention to stay [37,38]. This is particularly significant for nurses working in rural and remote communities [39,40]. Examples of extrinsic rewards include bonuses, raises, paid vacations, tuition reimbursement, and paid or unpaid leave to pursue further education [41-43]. Some countries have employed a number of strategies to improve the retention in rural areas, including financial incentives, trainings and continuing education programs and introducing supportive supervision and participatory management [7]. Nurse planners, managers and stakeholders are invited to offer an assortment of rewards targeting nurses working in rural communities in particular. Such rewards need not be expensive and their effects would need to be assessed in order to decide on the proper configuration that would enhance the satisfaction and retention of nurses in rural and remote communities [1,44].

While regression analysis reveals that working towards a higher degree negatively influenced nurse retention, the findings from the MMSS show that satisfaction with access to professional development opportunities had a positive impact on intent to stay. The consolidation of these two findings indicate that while working towards a higher degree might push rural nurses to seek a job in the more competitive urban areas (thus decreasing their intent to stay), providing opportunities for professional development through seminars, short courses and access to conferences (among others) would enhance the job retention of rural nurses. In that regards, the findings of this study are consistent with other studies in literature that indicate that rural nurses’ access to professional development and short term educational opportunities enhance their job satisfaction and support their retention [2,44,45]. Such programs are particularly important for health care providers working in rural and remote communities due to professional isolation and high cost of travel to acquire up to date knowledge [46]. Professional development programs were found to strengthen both the recruitment and retention of nurses in rural and remote areas, being valued even more to nurses than material rewards [1,29]. Establishing a continuing education and professional development program for nurses in underserved areas is a top priority for health policy and decision makers; particularly the order of nurses in Lebanon. Such programs do not only contribute to delivery of evidence based patient care but also have the potential to enhance nurses’ retention in rural and remote communities.

Study findings highlight differences between responses of nurses working in hospitals versus PHC centers. Nurses working in PHC centers were more likely to report an intent to stay. The literature points out some advantages to working in PHC centers in rural settings; mainly greater autonomy in caring for patients and ability to better utilize professional skills [2]. Furthermore, the shorter working hours in PHC centers might be a key reason for married nurses with children to prefer working in PHC centers particularly as evidence in the literature points out to nurses’ preference to work shorter hours [47]. This could also explain why more experienced nurses were more likely to work in PHC centers. Moreover nurses working in PHC centers had more positive attitudes towards nursing but they were less likely to report working towards a higher degree than nurses working in hospitals. While such findings are encouraging in regards to nurse retention within the expanding primary healthcare and community care network in Lebanon, they also highlight a policy priority to investigate and address the underlying causes behind nurse dissatisfaction with the hospital work environment in rural and remote communities in Lebanon. Identifying and addressing these causes would be a prerequisite to enhancing nurses’ job satisfaction and consequently retention in rural health facilities.

**Figure 1:**
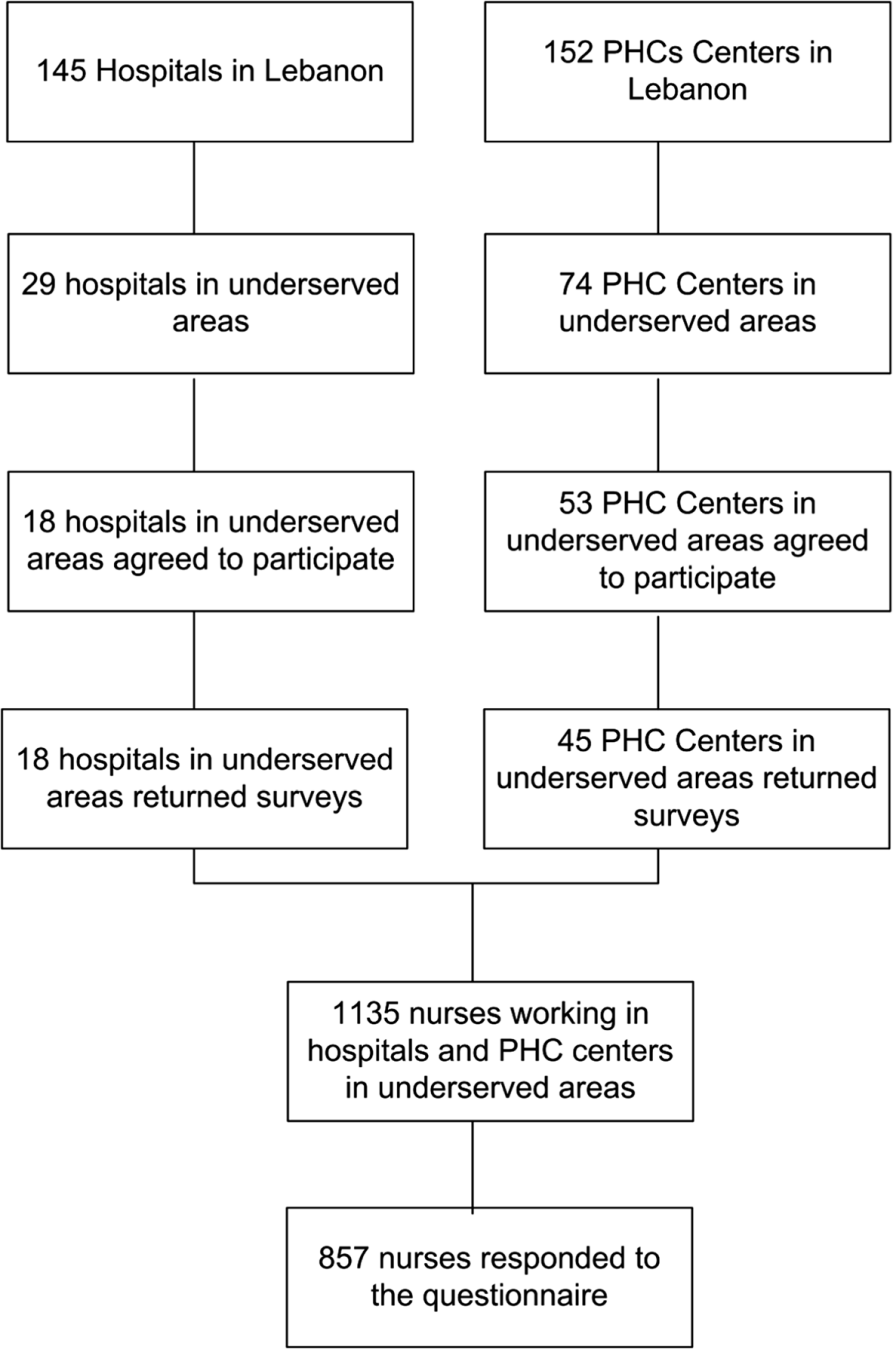
Data collection and response rate.

One of the main limitations for this study was the lack of information on the number and distribution of nurses across health facilities in Lebanon. The country also lacks clear cut boundaries that define rural areas from urban areas which made the mapping exercise a challenge. Having clearer boundaries and a more comprehensive database would have enabled a more thorough examination of the distribution of nurses in understaffed areas in Lebanon and allowed the authors to place the findings in the greater context of the Lebanese health care system. However, an extensive mapping of health facilities in the identified underserved areas was conducted to ensure that study findings represent the views of this stratum of the nursing workforce. The response rate could also be deemed one of the limitations of the study as we could not ascertain whether non-respondents carry a different opinion/experience than nurses that have responded to this study. However, the study had a relatively good response rate from nurses, with more than three quarters of targeted nurses (75.5%) responding to the questionnaire. In terms of facilities, and as portrayed in Figure 1, 62% of hospitals and 61% of PHC centers returned the surveys, indicating the majority of health facilities were able to participate in the study. Although these facilities were not different from the responding ones with regards to geographic location or the socioeconomic status of the catchment area, they tended to be smaller in size than the responding ones. The most common reasons for not participating in the study as reported by the facilities were: having too few or no nurses (particularly in PHC centers) or inability to designate a contact person at the facility to coordinate data collection. This indicates that the facilities may have been poorly staffed and lacked nursing personnel to complete the survey. Despite these challenges, we believe that the study had a very good response rate (75.5%) which enhances the external validity of the findings. An additional limitation relates to the inability of cross-sectional survey design to establish causality. However, the results of this manuscript outlines the association between factors influencing intent to stay which can provide lessons for decision makers on how to improve the available incentives for nurses in underserved areas. Finally, despite every attempt to ensure clarity of the questionnaire (pilot testing), it cannot be ascertained that all questions were well understood by nurses; especially among those with lower levels of education.

### Policy implications

The study findings highlighted major issues requiring attention from authorities at national, regional and international levels. Generating an accurate HRH data repository is pivotal in order to understand the supply, demand and distribution of HRH in general, and nurses in particular. Such a repository could utilize a Human Resource Information System to support evidence based HRH planning and management. Having an accurate account of nurse vacancies could help direct nurse supply (including unemployed nurses) to job opportunities in health facilities located in underserved and rural areas. Training of rural nursing cadre will also be needed not only to meet the specific health needs of the population residing in each underserved area, but also as a key motivator enhancing nurses’ retention in those areas. The Order of Nurses should assume a more proactive role in supporting, empowering, and educating practicing nurses. This is particularly important in light of the policy process that is currently underway to develop and implement the nursing profession practice law in Lebanon. A benefits and incentives system should be developed to encourage nurses to work in underserved areas and retain them. The Ministry of Health, Order of Nurses and Syndicates of Hospitals should collaborate in training administrators to assume a supportive role to nursing directors and nurses and give them more authority over staffing plans. Finally, education and research institutions should work collaboratively with the nursing stakeholders and decision makers in order to investigate and address the underlying causes for nurse dissatisfaction with the hospital work environment in rural and remote communities in Lebanon. Such collaboration would also be needed to design and implement targeted retention initiatives aimed at keeping younger nurses in rural and remote communities. Such initiatives must be designed in a participatory approach engaging younger nurses in planning and implementation.

## Conclusion

Study findings reflect a generally unstable and dissatisfied nursing workforce in rural and underserved areas in Lebanon. Such findings are disquieting to nursing planners, policy and decision makers as they cast a doubt on the sustainability and equity of providing health services to rural populations. Nevertheless, the study identifies multiple opportunities for intervention in order to rectify the situation and enhance the satisfaction and retention of the rural nursing workforce, with particular attention to the work setting.

Our findings highlight that the nursing workforce is not homogeneous, and nurses may want different things at different life stages and in different settings. Primary healthcare, often seen as the poorer and less desirable cousin of the higher status hospital sector, comes out rather well in this survey. The less demanding nature of the job in this sector meant that it was better able to retain nurses despite the relatively poor amenities. The issue of how hospitals in those underserved areas can create a contented workforce that is willing to commit to the longer term seems to be a much greater challenge. Stakeholders are urged to work collaboratively to devise strategies and initiatives that would bolster the retention of nurses in underserved areas; especially younger nurses and those working in the hospital. Such strategies would help enhance access, quality and equity of care delivered in rural settings and would contribute to improving the retention of the healthcare system’s most valuable asset…nurses.

## Abbreviations

BSN: Bachelor of Science in Nursing; BT: Baccalaureate technique; EMR: Eastern Mediterranean Region; ERC: Ethical review committee; HRH: Human resources for health; IRB: Institutional review board; LT: Licence technique; MS: Master of Nursing; MMSS: McCloskey Mueller satisfaction scale; NWI-R: Revised nurses work index; OR: Odds ration; PHC: Primary healthcare; TS: Technique superieur; WHO: World Health Organization.

## Competing interests

The authors declare that they have no competing interests.

## Authors’ contributions

FE contributed to the conception, study design, tool development, data collection, as well as data analysis and interpretation of results in addition to development of the manuscript. MA contributed to study conceptualization, tool development, interpretation of results and write up and revisions of the manuscript. DJ also contributed to study design, tool development, data analysis and interpretation and manuscript preparation. HD contributed to tool development, data analysis and interpretation, in addition to writing of the manuscript. NYD contributed to study design, tool development, data collection, and interpretation of results. MME contributed to tool development, and data collection. MJ contributed to tool development and data collection. SFM contributed to conception, study design, tool development, data analysis and interpretation of results and review of the manuscript. All authors read and approved the final manuscript.
